# Comprehensive genomic profiling of IgM multiple myeloma identifies *IRF4* as a prognostic marker

**DOI:** 10.18632/oncotarget.9478

**Published:** 2016-05-19

**Authors:** Daeun Ryu, Hee Jin Kim, Je-Gun Joung, Hae-Ock Lee, Joon Seol Bae, Seok Jin Kim, Haesu Kim, Woong-Yang Park, Kihyun Kim

**Affiliations:** ^1^ Samsung Genome Institute, Sungkyunkwan University School of Medicine, Seoul, Korea; ^2^ Department of Laboratory Medicine & Genetics, Sungkyunkwan University School of Medicine, Seoul, Korea; ^3^ Division of Hematology-Oncology, Department of Medicine, Samsung Medical Center, Sungkyunkwan University School of Medicine, Seoul, Korea; ^4^ Department of Health Sciences and Technology, Samsung Advanced Institute for Health Sciences and Technology, Sungkyunkwan University School of Medicine, Seoul, Korea; ^5^ Department of Molecular Cell Biology, Sungkyunkwan University School of Medicine, Seoul, Korea

**Keywords:** multiple myeloma, IgM, IRF4, progression-free survival, sequencing

## Abstract

Immunoglobulin M multiple myeloma (IgM MM) is an extremely rare subtype of multiple myeloma with a poor clinical outcome. In this study, bone marrow aspirates of MM patients, including two cases of IgM MM, were analyzed by whole exome sequencing and RNA sequencing. Recurrent somatic mutations in the *NRAS*, *KRAS*, *CCND1*, *DIS3*, and *TP53* genes were found in IgM MM and other types of MM, in agreement with previous studies. Overall transcription profiles of IgM and other types of MM clustered together, but separate from normal blood or peripheral plasma cells. Among the differentially expressed genes in IgM MM, *IRF4* was highly expressed in IgM as well as in a subset of other types of MM patients. Thus, *IRF4* is an independent prognostic factor for general MM patients. Taken together, the somatic mutation and transcriptome profiles support the idea that IgM MM can be classified as an aggressive MM subtype.

## INTRODUCTION

Multiple myeloma (MM) is a malignant disorder of bone marrow plasma cells, which typically produce large amounts of abnormal immunoglobulins during the course of the disease. The clinical manifestation of MM covers a broad spectrum ranging from asymptomatic to the mortality due to bone fractures, renal failure, and infectious complications [1]. Accumulating clinical and pathological evidence has led to improvements in the diagnosis of MM, and recently the International Myeloma Working Group updated its diagnosis criteria [2]. With regard to the types of abnormal immunoglobulin produced by malignant plasma cells in MM, IgG accounts for about half of the cases, followed by IgA (~20%). Multiple myeloma producing IgM (IgM MM) is a very rare (0.1-0.5%) and aggressive disease [3].

IgM MM must be distinguished from other IgM-producing plasma cell disorders, particularly Waldenström's macroglobulinemia (WM) as the two diseases have different treatment options [4]. Recent advancements in the molecular techniques revealed genomic characteristics of MM and WM [5, 6], and the unique genetic alterations found in WM enabled otherwise challenging differential diagnoses between the two diseases [7, 8]. However, the genomic characteristics of IgM MM have been partially defined by low throughput molecular studies, and comprehensive genomic profiling has been lacking. In this study, we investigated genomic characteristics of IgM MM by analyzing whole exome and whole transcriptome sequencing data for two IgM MM cases in relation to other types of MM. Whole exome sequencing (WES) identified recurrent DNA aberrations in *DIS3* and *MYO10* for IgM MM, which were found in other MM cases at low frequencies. The transcriptome profiling also allowed classification of IgM MM as an MM subgroup, and identified *IRF4* as a prognostic marker shared by IgM-type and other types of MM with aggressive disease progression.

## RESULTS

### Somatic mutation profiles of IgM MM

**Figure 1:**
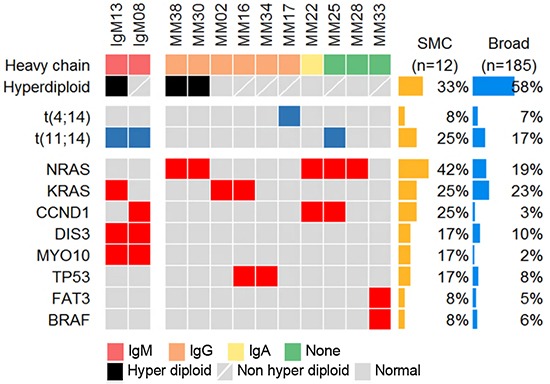
Mutational landscape of multiple myelomas (MMs), including the detected mutations from IgM MM patients Heat map describing the recurrent somatic alterations in MM. The mutation frequency is obtained from our sequencing data (SMC) and Broad [9], (which was downloaded from cBioPortal).

We performed whole exome sequencing (WES) for CD138+ enriched bone marrow cells from two IgM MM patients and 10 other types of MM (Supplementary Table 2). The average sequencing depth for WES was 130.0X (± 18.23). Sequencing reads covered whole exome regions with at least 98.5% (over 10X). Recurrent somatic single nucleotide variations (SNVs) in 12 MM patients including two IgM MM patients were identified from our study (Figure 1). In particular, SNVs in *DIS3* and *MYO10*, reported at frequencies of 10% and 2% in MM [9], were found in both IgM MM patients. The *DIS3* gene had missense mutations at the same amino acid residue (R780) with different base substitutions. Two mutations, K1767M and Y1668C in *MYO10*, were located between the Myosin Tail Homology 4 (MyTH4) domain and the FERM central domain. Other interesting mutations occurred in *CCND1* (S55Y, in the first exon) for IgM08 and *KRAS* (G12V) for IgM13. *CCND1* mutations in the first exon and the accompanying chromosomal translocation t(11;14) may indicate ongoing somatic hypermutation driven by activation-induced cytidine deaminase (AID) protein [9, 10]. The *KRAS* G12V mutation is known as a driver mutation of MMs as well as other cancers. Notably, the *MYD88* L265P mutation, associated with Waldenström's macroglobulinemia, was detected in none of the MM samples. We performed Sanger sequencing for the three genes (*DIS3*, *MYO10*, and *MYD88*) and verified WES results (Supplementary Figure S1).

### Chromosomal abnormalities in IgM MM

**Figure 2:**
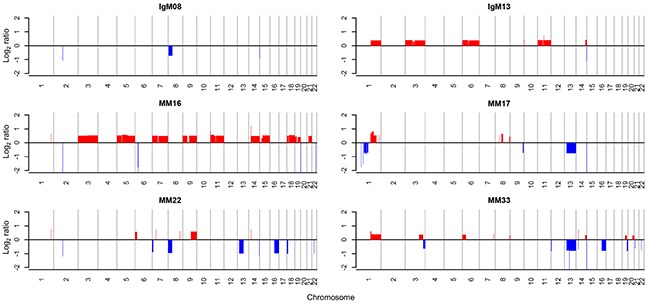
Copy number alterations of MM patients Red and blue colors indicate amplification and deletion, respectively

According to previous studies, approximately 14% of whole MM samples harbor the chromosomal translocation t(11;14) [11], whereas 7 out of 8 IgM MM showed t(11;14) in cytogenetic analyses [12]. The two IgM MM samples in the current study also harbored t(11;14) as analyzed by fluorescence *in situ* hybridization (FISH), supporting the high incidence of t(11;14) in IgM MM (Figure 1). In addition, copy number variation (CNV) analysis indicated trisomy of chromosomes 1q, 3, 6, and 11 as well as focal amplification of 14q32.33 in the hyperdiploid IgM MM (Figure 2). The overall pattern of CNV in the IgM MM samples was similar to the previously observed pattern in other MMs.

### Comparison of gene expression profiles to other types of MMs

**Figure 3:**
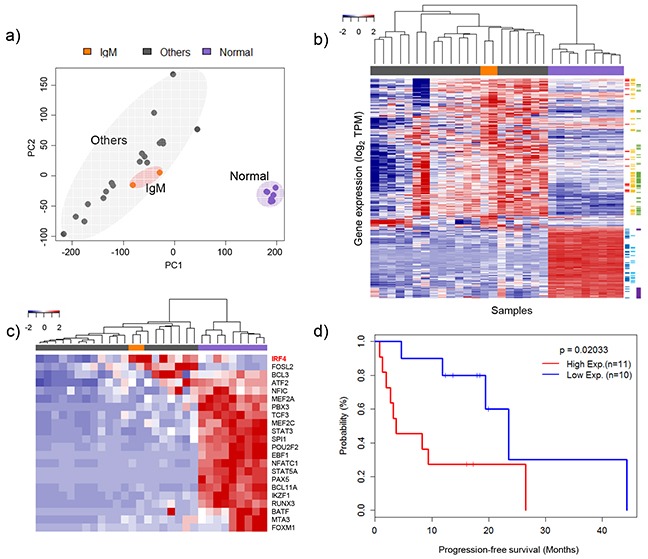
Clustering of gene expression profiles and heat map for specific gene sets **a.** Principle component analysis of gene expression levels. Three distinct groups were plotted in two-dimensional space: IgM types, other types (IgG, IgA, and no heavy chain) and normal samples. **b.** Clustering of differentially expressed genes. Red, black, and blue groups indicate IgM types, other types and normal samples, respectively. Sidebar is color coded by molecular function using David (https://david.ncifcrf.gov): red = oxidative phosphorylation, yellow = organelle membrane, green = ER related genes, blue = immune response, sky-blue = leukocyte activation and purple = immunoglobulins. **c.** Heat map of the expression profiles of genes associated with B-cell-specific transcription factors (TFs) [14]. **d.** Clinical outcomes of multiple myeloma (MM) patients according to *IRF4* expression level.

RNA sequencing was performed on 21 MM samples including the twelve with WES analysis. Across all RNA-Seq data, 45.85 ± 1.05% of total reads were uniquely aligned to the human genome reference. The transcriptome analysis focused on the similarities and differences of gene expression patterns between IgM and the other types of MM. The expression pattern of IgM and other types of MM is markedly different from normal control cells (Figure 3a). IgM MM samples clustered with the group of MM samples, suggesting its inclusion in the MM subgroup. When we clustered them with 618 differentially expressed genes (DEGs) between IgM MM and normal control cells, most of the MM samples showed a similar expression pattern. Nevertheless, IgM MM could be grouped separately from the other MM types, showing subtle differences in gene expression (Figure 3b and Supplementary Table 3). Compared to the normal control cells, underexpressed genes were more prevalent than overexpressed genes, especially those related to major histocompatibility complex (MHC) class II and B-cell activation or differentiation (Supplementary Table 3). Overexpressed genes belonged to endoplasmic reticulum (ER) or mitochondrial components, which are elevated in MM in general [13]. A recent report suggested that MMs have distinct methylation and gene expression status for the B-cell-specific transcription factors (TFs) [14]. Interestingly, interferon regulatory factor 4 (IRF4), an indispensable transcription factor for plasma cell differentiation, was overexpressed in both IgM MM samples (Figure 3c). IRF4 is also known to be a survival factor for MM cells and correlates with the aggressive disease status [15, 16]. In our dataset, IRF4 also showed high expression levels in the most aggressive MMs as well as in the IgM MMs (Figure 3d and Supplementary Figure S2). The high-expression group showed shorter progression-free time (p =0.020) compared to the low-expression group. When we took other clinical parameters such as age, International Staging System (ISS) Stage, levels of lactate dehydrogenase, and high risk factors (HR; t(4;14) and 17p deletion [17]) into account, the high risk group showed poorer outcome (p = 0.05, Supplementary Figure S4), suggesting that IRF4 is an independent prognostic factor. We used independent public datasets to evaluate the prognostic significance of *IRF4* expression and found a positive correlation (Supplementary Figure S3). The tumorigenesis mechanisms involving IRF4 are currently unclear, but its association in MM development and disease progression warrants further investigation.

## DISCUSSION

In this study, we performed comprehensive genomic profiling of IgM MM, which is an aggressive MM subtype with a low incidence rate of < 0.5% [18, 19]. Our results show that features of the IgM type were similar to the known genomic characteristics of MMs.

Several genes recurrently mutated in MM, such as *KRAS*, *NRAS*, *FAM46C*, *DIS3*, *TP53*, and *BRAF*, have been reported [9, 20]. Among those, mutation of *DIS3* at position R780 was detected in both IgM MM samples. The DIS3 protein is an RNA exosome subunit containing exonucleolytic (RNB) and endonucleolytic (PIN) domains, and participates in the regulation of mRNA and small RNA processing [21–23]. The R780 substitution mutation in the RNB domain eliminates the exonucleolytic activity of DIS3 [24, 25], and thus may cause perturbations in cellular RNA metabolism and gene expression. Recently, Robinson et al. [26] suggested additional effects of *DIS3* mutation in MM, i.e., defects in spindle assembly checkpoint and/or aberrant hypermutation and antibody class switching. These defects may cause genetic instability and aneuploidy, which are frequently found in multiple myeloma. As our IgM MM cases include one with aneuploidy (hyperdiploidy) and one with normal cytogenetics, the oncogenic effect of DIS3 mutation may be exerted by multiple mechanisms.

In the transcriptional study, we found that IRF4, an interferon regulatory factor and B-cell specific transcription factor, is a prognostic factor in multiple myeloma independent of patient age, ISS disease stage, levels of lactate dehydrogenase, and high risk factors. Previously, IRF4 positivity in immunohistochemical staining had a significant correlation with increased disease stage in multiple myeloma [15], and its silencing caused cell death in myeloma cell lines [16]. Through gene expression profiling and chromatin immunoprecipitation analysis [16], the Myc oncogene was demonstrated as a direct target of IRF4, and IRF4 was proposed as a master regulator of malignancy-specific gene expression in multiple myeloma.

Several cytogenetic abnormalities have been proposed as poor prognostic factors in multiple myeloma, such as the chromosome translocations t(4;14) and t(14;16), non-hyperdiploidy, 1q gains and 17p deletions [27]. In addition, gene expression signatures from 70 prognostic genes are being developed to predict the risk of relapse for MM patients [28]. By comparison, only a few genetic aberrations at the single gene level, such as *DIS3* mutation [29] and *IRF4* overexpression [15] were proposed as a poor prognostic factor. As we identified *DIS3* mutation and *IRF4* overexpression in IgM MM, these aberrations may contribute to the aggressive disease phenotype in this rare MM subtype.

## MATERIALS AND METHODS

### Patients and sample preparation

This study was approved by the institutional review board (IRB) of Samsung Medical Center (IRB approval no. SMC2013-09-009-012) and carried out in accordance with the principles of the Declaration of Helsinki. The study subjects were 21 Korean patients diagnosed with multiple myeloma including two with IgM MM at Samsung Medical Center, Seoul, Korea (Supplementary Table S1). Bone marrow aspirates after initial diagnosis were subjected to Ficoll-Paque PLUS (GE Healthcare, USA) gradient and magnetic separation with anti-CD138 antibody microbeads (Miltenyi Biotech, Germany). From the CD138-enriched bone marrow cells, genomic DNA and RNA was purified using the ALLPrep kit (Qiagen, USA). Matching blood DNA was isolated by the QIAamp DNA blood kit (Qiagen). RNAs from 21 patients were subjected to RNA sequencing whereas only 12 DNA samples were subjected to whole exome sequencing. Normal control RNA was collected from CD19+ blood B cells from four healthy volunteers and CD138+ tonsil plasma cells from five tonsilectomy tissues.

### Whole exome sequencing and data analysis

Genomic DNA (1 μg) from the bone marrow and matching blood samples was sheared by Covaris S220 (Covaris, MA, USA) and used for library construction with SureSelect XT Human All Exon v5 and SureSelect XT reagent kit, HSQ (Agilent Technologies, Santa Clara, CA, USA) according to manufacturer's protocols. After multiplexing, the libraries were sequenced on the HiSeq 2500 sequencing platform (Illumina, USA), using the 100 bp paired-end mode of the TruSeq Rapid PE Cluster kit and TruSeq Rapid SBS kit (Illumina).

Sequencing reads were aligned to the UCSC hg19 reference genome (downloaded from http://genome.ucsc.edu) using Burrows-Wheeler Aligner (BWA) [30], version0.6.2 with default settings. PCR duplications are marked by Picard-tools-1.8 (http://picard.sourceforge.net/), data cleanup was followed by GATK, and variants were identified with GATK-2.2.9 (https://www.broadinstitute.org/gatk/). Then, point mutations were identified by MuTect (https://github.com/broadinstitute/mutect) and VarScan 2 (http://varscan.sourceforge.net) with paired samples. Perl script and ANNOVAR [31] were used to annotate variants.

### RNA sequencing and data analysis

The library construction for whole transcriptome sequencing was performed using the TruSeq RNA sample preparation v2 kit (Illumina). Sequencing of the transcriptome library was carried out using the 100 bp paired-end mode of the TruSeq Rapid PE Cluster kit and TruSeq Rapid SBS kit (Illumina).

The reads from the FASTQ files were mapped against the GRCh37.75 human reference genome by using STAR (https://github.com/alexdobin/STAR/releases) version 2.4.0. The output files in BAM format were analyzed by RSEM (http://deweylab.biostat.wisc.edu/rsem/) version 1.2.18 to quantify the transcript abundance in transcripts per million (TPM). Coding genes were selected (20,652) and low-expression genes were filtered out by applying the criteria that the total TPM should be > 20.42 (mean TPM value) across all samples. Clustering was performed by Principal Component Analysis (PCA). We identified differentially expressed genes (DEGs) and performed gene ontology (GO) analysis using the ‘DESeq’ [32] which is Bioconductor package (http://bioconductor.org) in R and ‘DAVID’ [33]. We used two GEO datasets to evaluate the prognostic significance of *IRF4* expression (GSE9782 [34], GSE24080 [35]).

### Validation of SNVs by Sanger sequencing

Regions flanking the mutation sites of *DIS3* and *MYO10* were amplified from genomic DNA and subjected to Sanger sequencing. The primer sequences are DIS3 forward: 5′-TTAGCCACTCGCTGTATGATG-3′; DIS3 reverse: 5′-G ATGCACGTTGGGCATATTG-3′; DIS3 sequencing: 5′-GC TTGTGTTTGTCTGTCAACTC-3′; MYO10(5003) forward: 5′-CAGCAAAGGCATCTAACAGAAC-3′; MYO10(5003) reverse: 5′-AGTGAGACATTGGCTCTTTAGG-3′; MYO10 (5003) sequencing: 5′-GTGGGAGTTGATGGTGATCTT-3′; MYO10(5300) forward: 5′-TCACGTAAGACCGTAGCT TTATC-3′; MYO10(5300) reverse: 5′-GATGACCACAC GGGTAACAT-3′; MYO10(5300) sequencing: 5′-ACAG GCATCAACACAGGTAAA-3′. Regions flanking the WM hotspot mutation site MYD88 L265 were amplified and sequenced by MEMO (Mutant Enrichment with 3′-Modified Oligonucleotides) PCR [36] using primers MYD88 blocking: 5′-AAGCGACTGATCCCCATCAA-3′[C3SP]; MYD88 forward: 5′-CAGGTGCCCATCAGAAGC-3′; MYD88 reverse: 5′-GAAGTTGGCATCTCCAGGAA-3′. MYD88 reverse primer was used as a sequencing primer.

### Real time RT-PCR analysis

Three nanogram of total RNA was used for the RT-PCR reaction using One-step RT-PCR premix kit (iNtRON, Seoul, Korea) with SYBR Green for IRF4 and GAPDH. Primer sequences are IRF4 forward: 5′-CCCGGAAATCCCGTACCAAT-3′; IRF4 reverse: 5′-AGGTGGGGCACAAGCATAAA-3′; GAPDH forward: 5′-GAAGGTGAAGGTCGGAGT-3′; GAPDH reverse: 5′-TGGCAACAATATCCACTTTACCA-3′. Normalized *IRF4* mRNA expression is presented as −ΔCt (IRF4-GAPDH).

## SUPPLEMENTARY FIGURES AND TABLES








