# Supplementary material for: Comprehensive genomic profiling of IgM multiple myeloma identifies IRF4 as a prognostic marker
Source: Oncotarget. 2016 May 19;7(30):47127–33. doi: 10.18632/oncotarget.9478 (PMC5216929; doi:10.18632/oncotarget.9478)
Supplement: Supplementary file 1 [file oncotarget-07-47127-s001.pdf]

Comprehensive genomic profiling of IgM multiple myeloma identifies *IRF4* as a prognostic marker

SUPPLEMENTARY FIGURES AND TABLES

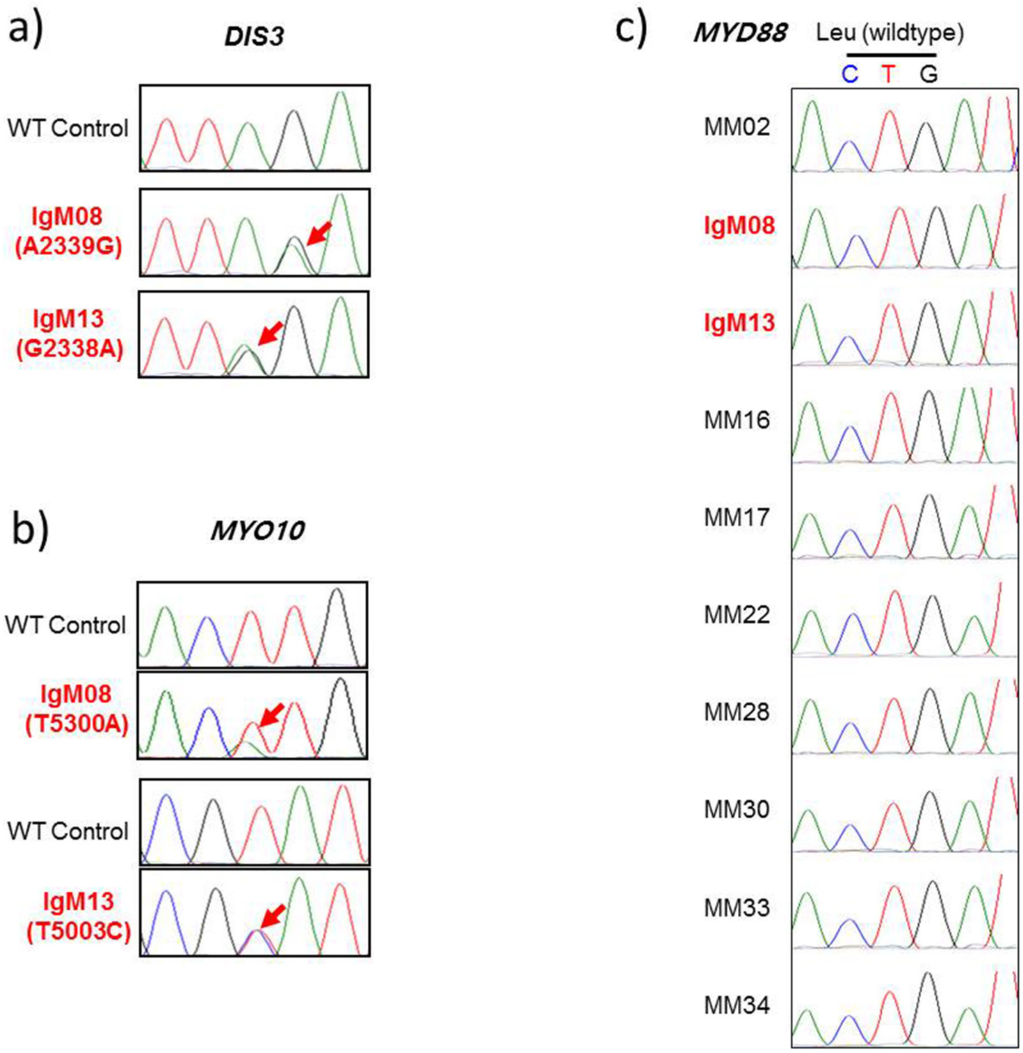

Supplementary Figure S1: Validations for mutations by sanger sequencing. Detected mutations from WES were confirmed in **a.** *DIS3* and **b.** *MYO10*. **c.** Wild-type *MYD88* in the L265 Waldenstrom's macroglobulinemia hotspot mutation site in MM samples.

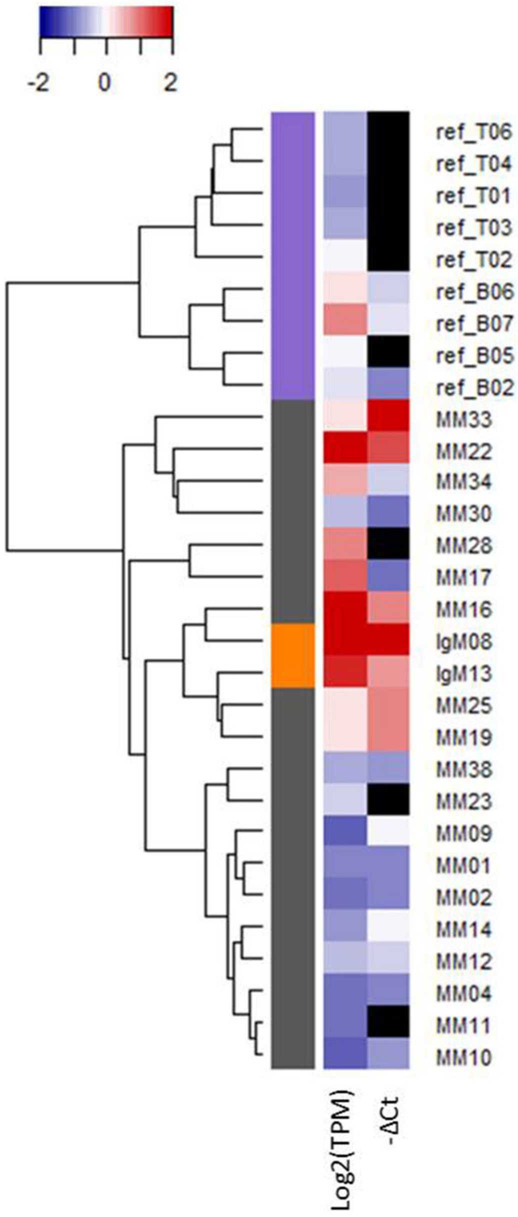

Supplementary Figure S2: IRF4 expression by real-time RT-PCR. IRF4 expression was confirmed by real-time RT-PCR in 21/30 samples. Side-bar indicates sample type : orange = IgM, gray = other type of MM, purple = normal. Black in heatmap is not available sample for real-time PCR.

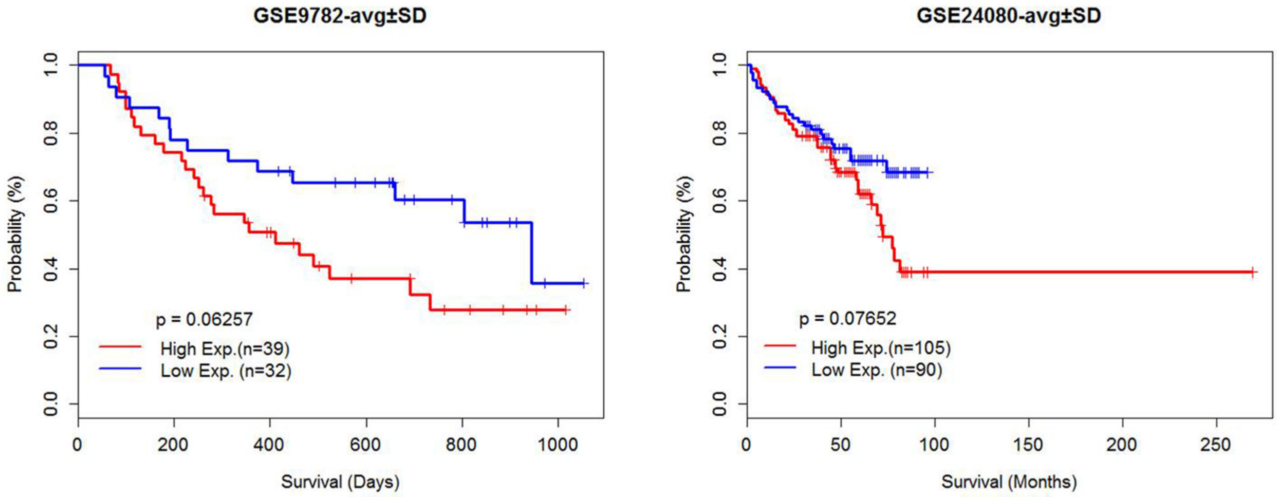

Supplementary Figure S3: Survival analysis using public data-set. The prognostic significance of IRF4 expression was evaluated in 2 public datasets.IRF4 high-expression group has IRF4 expression over the [sum of average and standard deviation], and the low-expression group under [the sum of average and negative standard deviation].

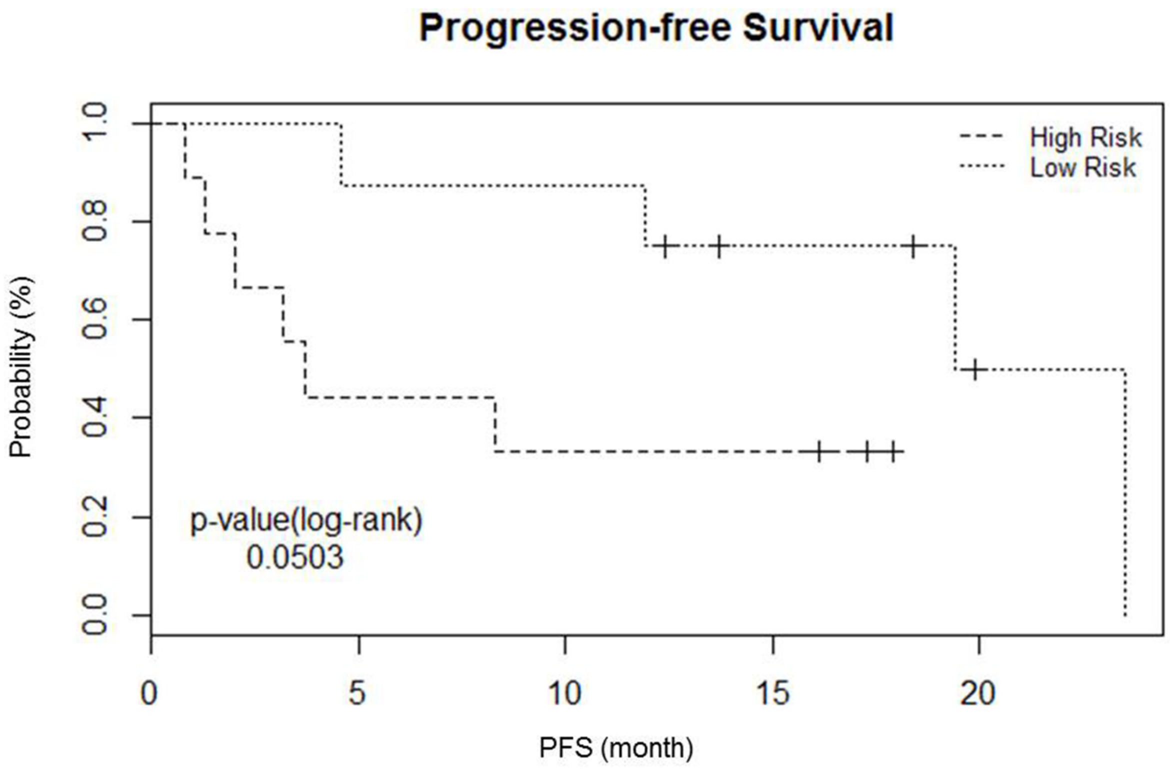

Adj. parameter : Age, ISS, LD, HR, IRF4exp.

	Hazard ratio	p-value	95% CI
**Age**	1.01304	0.669	0.95462 - 1.075
**ISS**	1.50262	0.4326	0.54351 - 4.1543
**LD**	1.42865	0.7191	0.20455 - 9.978
**HR**	6.33172	0.0966	0.71811 - 55.8284
**IRF4 exp.**	0.10314	0.0231*	0.01454 - 0.7316

Supplementary Figure S4: IRF4 is an independent prognostic factor. Cox's proportional hazard model was applied to our data to test IRF4 as an independent prognostic factor. Considered clinical parameters were age, International Staging System (ISS) Stage, levels of lactate dehydrogenase (LD), and high risk factors (t(4;14) and 17p deletion).

Supplementary Table S1: Summary of clinical features

See Supplementary File 1

Supplementary Table S2: List of detected mutations

See Supplementary File 2

Supplementary Table S3: The results of differential expressed gene analysis between IgM MM and the normal samples

See Supplementary File 3
